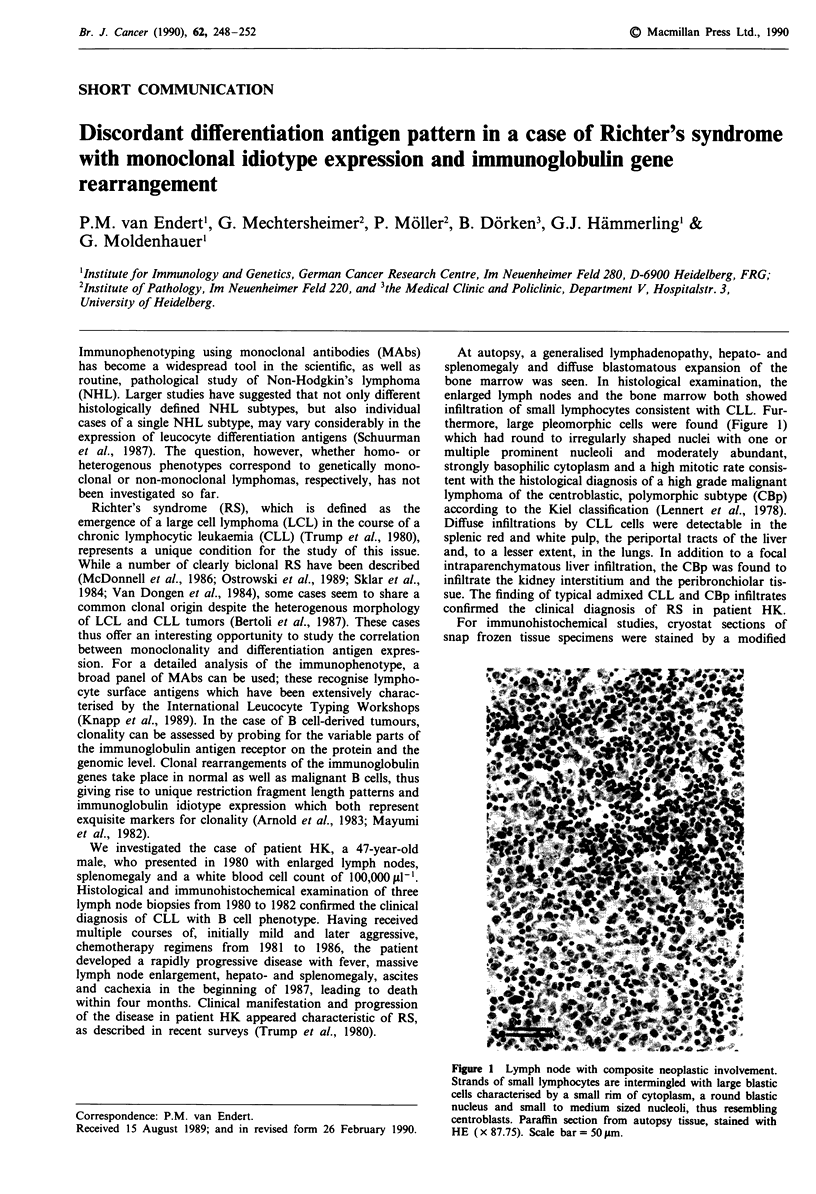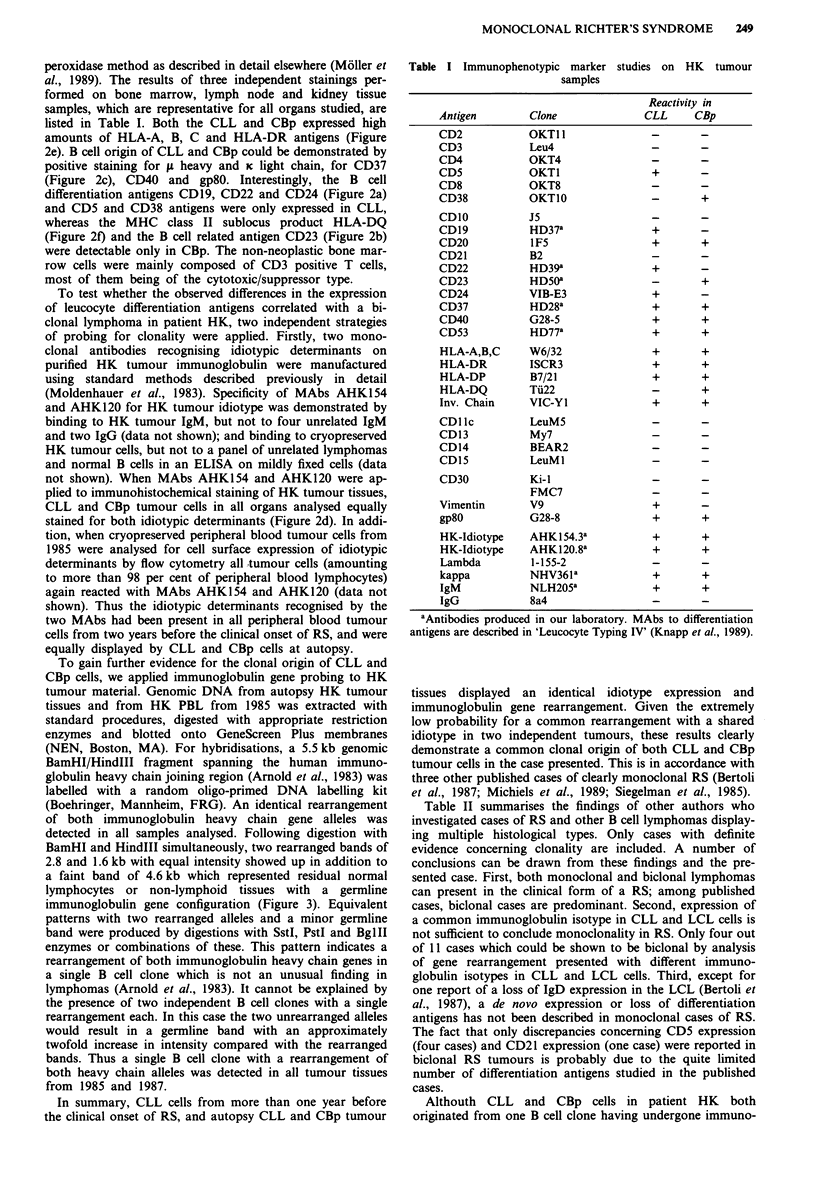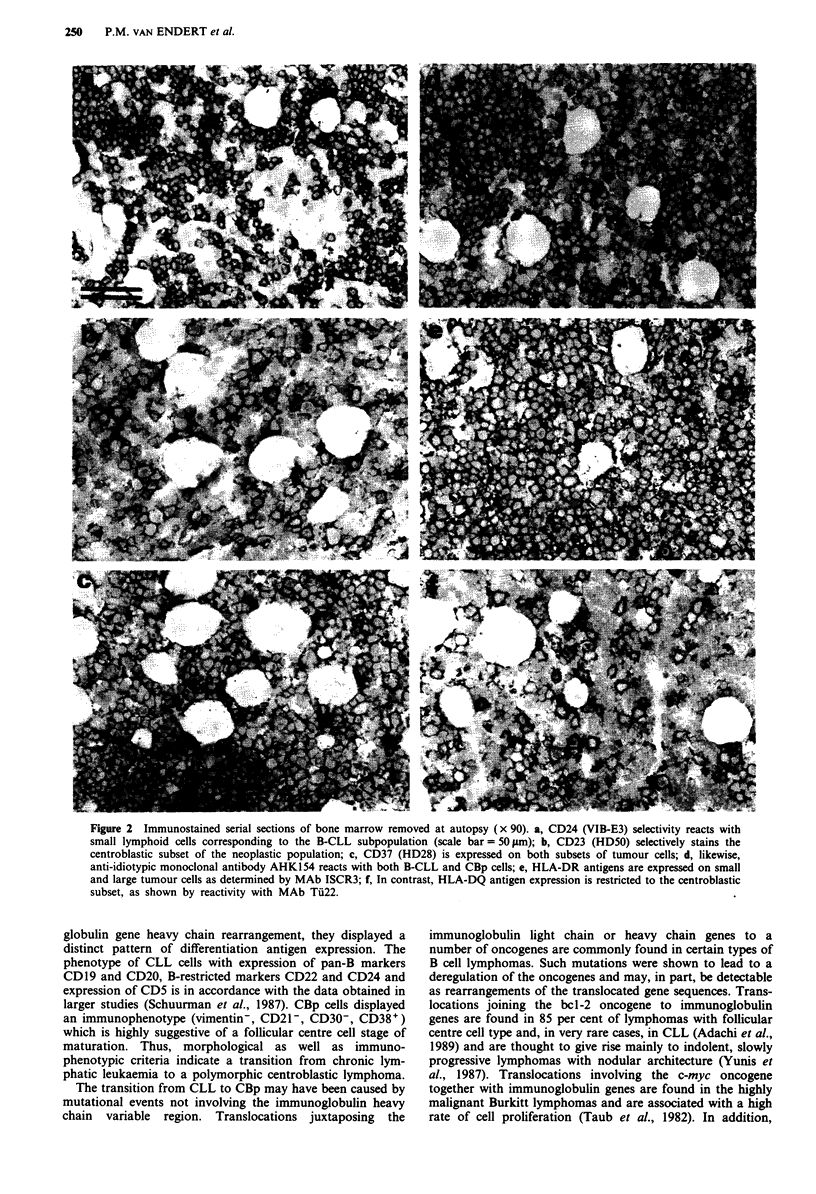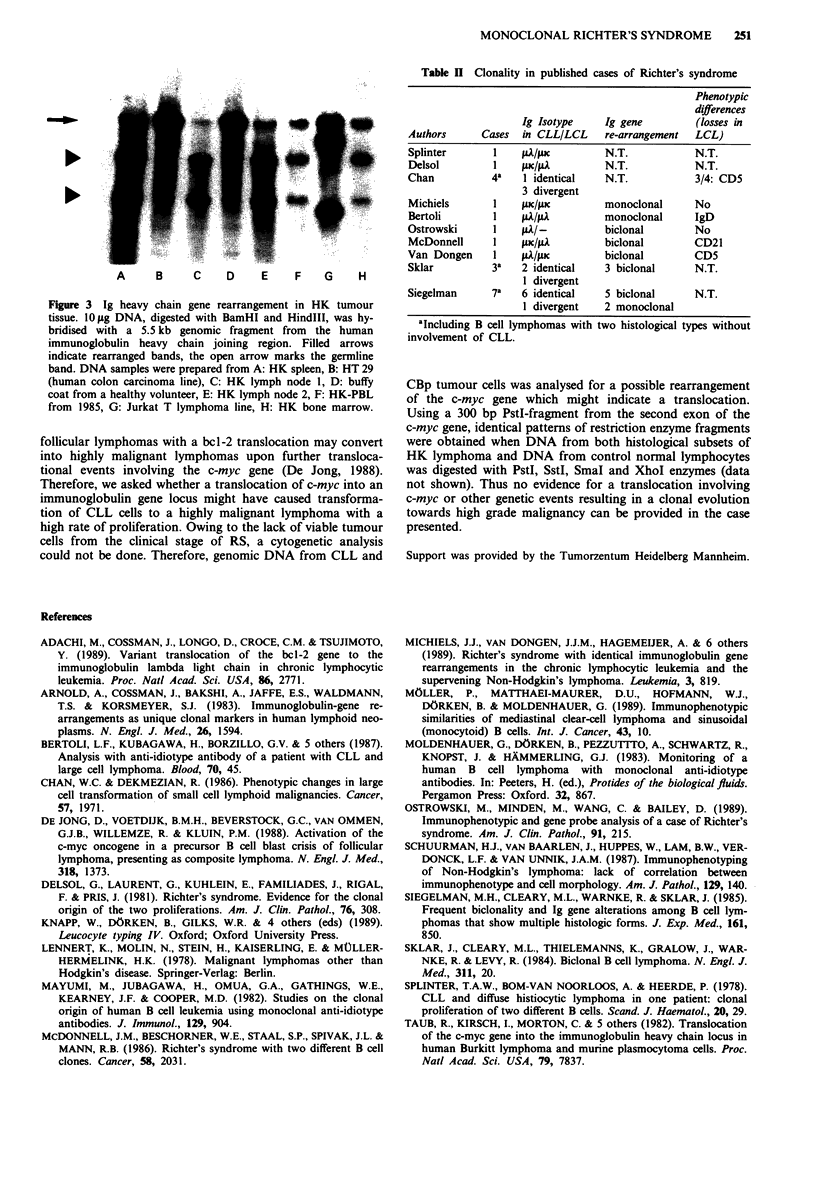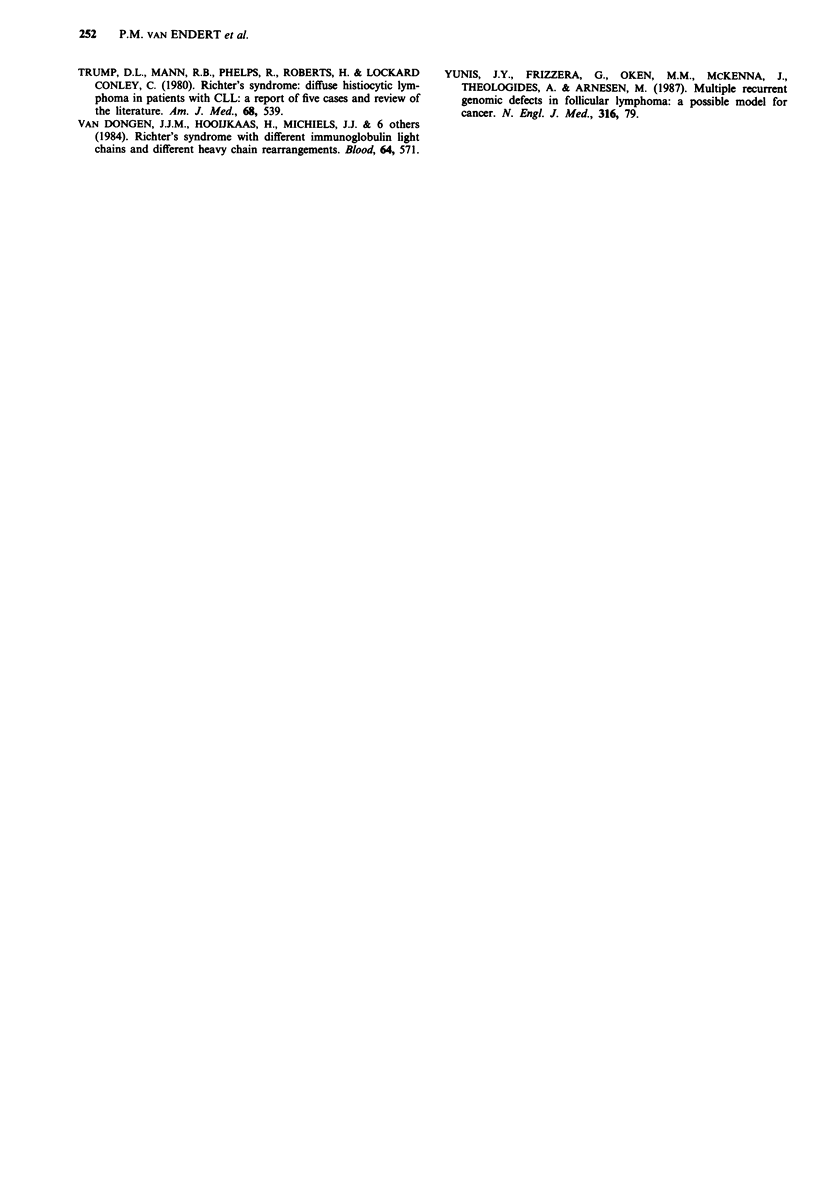# Discordant differentiation antigen pattern in a case of Richter's syndrome with monoclonal idiotype expression and immunoglobulin gene rearrangement.

**DOI:** 10.1038/bjc.1990.269

**Published:** 1990-08

**Authors:** P. M. van Endert, G. Mechtersheimer, P. Möller, B. Dörken, G. J. Hämmerling, G. Moldenhauer

**Affiliations:** Institute for Immunology and Genetics, German Cancer Research Centre, Heidelberg.

## Abstract

**Images:**


Br. J. Cancer (1990), 62, 248-252 © Macmillan Press Ltd., 1990

SHORT COMMUNICATION

### Discordant differentiation antigen pattern in a case of Richter's syndrome with monoclonal idiotype expression and immunoglobulin gene rearrangement

P.M. van Endert[1], G. Mechtersheimer[2], P. Möller[2], B. Dörken[3], G.J. Hämmerling[1] & G. Moldenhauer[1]

[1]*Institute for Immunology and Genetics, German Cancer Research Centre, Im Neuenheimer Feld 280, D-6900 Heidelberg, FRG;* [2]*Institute of Pathology, Im Neuenheimer Feld 220, and* [3]*the Medical Clinic and Policlinic, Department V, Hospitalstr. 3, University of Heidelberg.*

Immunophenotyping using monoclonal antibodies (MAbs) has become a widespread tool in the scientific, as well as routine, pathological study of Non-Hodgkin's lymphoma (NHL). Larger studies have suggested that not only different histologically defined NHL subtypes, but also individual cases of a single NHL subtype, may vary considerably in the expression of leucocyte differentiation antigens (Schuurman *et al.*, 1987). The question, however, whether homo- or heterogenous phenotypes correspond to genetically monoclonal or non-monoclonal lymphomas, respectively, has not been investigated so far.

Richter's syndrome (RS), which is defined as the emergence of a large cell lymphoma (LCL) in the course of a chronic lymphocytic leukaemia (CLL) (Trump *et al.*, 1980), represents a unique condition for the study of this issue. While a number of clearly biclonal RS have been described (McDonnell *et al.*, 1986; Ostrowski *et al.*, 1989; Sklar *et al.*, 1984; Van Dongen *et al.*, 1984), some cases seem to share a common clonal origin despite the heterogenous morphology of LCL and CLL tumors (Bertoli *et al.*, 1987). These cases thus offer an interesting opportunity to study the correlation between monoclonality and differentiation antigen expression. For a detailed analysis of the immunophenotype, a broad panel of MAbs can be used; these recognise lymphocyte surface antigens which have been extensively characterised by the International Leucocyte Typing Workshops (Knapp *et al.*, 1989). In the case of B cell-derived tumours, clonality can be assessed by probing for the variable parts of the immunoglobulin antigen receptor on the protein and the genomic level. Clonal rearrangements of the immunoglobulin genes take place in normal as well as malignant B cells, thus giving rise to unique restriction fragment length patterns and immunoglobulin idiotype expression which both represent exquisite markers for clonality (Arnold *et al.*, 1983; Mayumi *et al.*, 1982).

We investigated the case of patient HK, a 47-year-old male, who presented in 1980 with enlarged lymph nodes, splenomegaly and a white blood cell count of 100,000 $\mu l^{-1}$. Histological and immunohistochemical examination of three lymph node biopsies from 1980 to 1982 confirmed the clinical diagnosis of CLL with B cell phenotype. Having received multiple courses of, initially mild and later aggressive, chemotherapy regimens from 1981 to 1986, the patient developed a rapidly progressive disease with fever, massive lymph node enlargement, hepato- and splenomegaly, ascites and cachexia in the beginning of 1987, leading to death within four months. Clinical manifestation and progression of the disease in patient HK appeared characteristic of RS, as described in recent surveys (Trump *et al.*, 1980).

At autopsy, a generalised lymphadenopathy, hepato- and splenomegaly and diffuse blastomatous expansion of the bone marrow was seen. In histological examination, the enlarged lymph nodes and the bone marrow both showed infiltration of small lymphocytes consistent with CLL. Furthermore, large pleomorphic cells were found (Figure 1) which had round to irregularly shaped nuclei with one or multiple prominent nucleoli and moderately abundant, strongly basophilic cytoplasm and a high mitotic rate consistent with the histological diagnosis of a high grade malignant lymphoma of the centroblastic, polymorphic subtype (CBp) according to the Kiel classification (Lennert *et al.*, 1978). Diffuse infiltrations by CLL cells were detectable in the splenic red and white pulp, the periportal tracts of the liver and, to a lesser extent, in the lungs. In addition to a focal intraparenchymatous liver infiltration, the CBp was found to infiltrate the kidney interstitium and the peribronchiolar tissue. The finding of typical admixed CLL and CBp infiltrates confirmed the clinical diagnosis of RS in patient HK.

For immunohistochemical studies, cryostat sections of snap frozen tissue specimens were stained by a modified

**Figure 1** Lymph node with composite neoplastic involvement. Strands of small lymphocytes are intermingled with large blastic cells characterised by a small rim of cytoplasm, a round blastic nucleus and small to medium sized nucleoli, thus resembling centroblasts. Paraffin section from autopsy tissue, stained with HE (×87.75). Scale bar = 50 μm.

Correspondence: P.M. van Endert.
Received 15 August 1989; and in revised form 26 February 1990.

peroxidase method as described in detail elsewhere (Möller *et al.*, 1989). The results of three independent stainings performed on bone marrow, lymph node and kidney tissue samples, which are representative for all organs studied, are listed in Table I. Both the CLL and CBp expressed high amounts of HLA-A, B, C and HLA-DR antigens (Figure 2e). B cell origin of CLL and CBp could be demonstrated by positive staining for μ heavy and κ light chain, for CD37 (Figure 2c), CD40 and gp80. Interestingly, the B cell differentiation antigens CD19, CD22 and CD24 (Figure 2a) and CD5 and CD38 antigens were only expressed in CLL, whereas the MHC class II sublocus product HLA-DQ (Figure 2f) and the B cell related antigen CD23 (Figure 2b) were detectable only in CBp. The non-neoplastic bone marrow cells were mainly composed of CD3 positive T cells, most of them being of the cytotoxic/suppressor type.

To test whether the observed differences in the expression of leucocyte differentiation antigens correlated with a biclonal lymphoma in patient HK, two independent strategies of probing for clonality were applied. Firstly, two monoclonal antibodies recognising idiotypic determinants on purified HK tumour immunoglobulin were manufactured using standard methods described previously in detail (Moldenhauer *et al.*, 1983). Specificity of MAbs AHK154 and AHK120 for HK tumour idiotype was demonstrated by binding to HK tumour IgM, but not to four unrelated IgM and two IgG (data not shown); and binding to cryopreserved HK tumour cells, but not to a panel of unrelated lymphomas and normal B cells in an ELISA on mildly fixed cells (data not shown). When MAbs AHK154 and AHK120 were applied to immunohistochemical staining of HK tumour tissues, CLL and CBp tumour cells in all organs analysed equally stained for both idiotypic determinants (Figure 2d). In addition, when cryopreserved peripheral blood tumour cells from 1985 were analysed for cell surface expression of idiotypic determinants by flow cytometry all tumour cells (amounting to more than 98 per cent of peripheral blood lymphocytes) again reacted with MAbs AHK154 and AHK120 (data not shown). Thus the idiotypic determinants recognised by the two MAbs had been present in all peripheral blood tumour cells from two years before the clinical onset of RS, and were equally displayed by CLL and CBp cells at autopsy.

To gain further evidence for the clonal origin of CLL and CBp cells, we applied immunoglobulin gene probing to HK tumour material. Genomic DNA from autopsy HK tumour tissues and from HK PBL from 1985 was extracted with standard procedures, digested with appropriate restriction enzymes and blotted onto GeneScreen Plus membranes (NEN, Boston, MA). For hybridisations, a 5.5 kb genomic BamHI/HindIII fragment spanning the human immunoglobulin heavy chain joining region (Arnold *et al.*, 1983) was labelled with a random oligo-primed DNA labelling kit (Boehringer, Mannheim, FRG). An identical rearrangement of both immunoglobulin heavy chain gene alleles was detected in all samples analysed. Following digestion with BamHI and HindIII simultaneously, two rearranged bands of 2.8 and 1.6 kb with equal intensity showed up in addition to a faint band of 4.6 kb which represented residual normal lymphocytes or non-lymphoid tissues with a germline immunoglobulin gene configuration (Figure 3). Equivalent patterns with two rearranged alleles and a minor germline band were produced by digestions with SstI, PstI and BglII enzymes or combinations of these. This pattern indicates a rearrangement of both immunoglobulin heavy chain genes in a single B cell clone which is not an unusual finding in lymphomas (Arnold *et al.*, 1983). It cannot be explained by the presence of two independent B cell clones with a single rearrangement each. In this case the two unrearranged alleles would result in a germline band with an approximately twofold increase in intensity compared with the rearranged bands. Thus a single B cell clone with a rearrangement of both heavy chain alleles was detected in all tumour tissues from 1985 and 1987.

In summary, CLL cells from more than one year before the clinical onset of RS, and autopsy CLL and CBp tumour tissues displayed an identical idiotype expression and immunoglobulin gene rearrangement. Given the extremely low probability for a common rearrangement with a shared idiotype in two independent tumours, these results clearly demonstrate a common clonal origin of both CLL and CBp tumour cells in the case presented. This is in accordance with three other published cases of clearly monoclonal RS (Bertoli *et al.*, 1987; Michiels *et al.*, 1989; Siegelman *et al.*, 1985).

Table II summarises the findings of other authors who investigated cases of RS and other B cell lymphomas displaying multiple histological types. Only cases with definite evidence concerning clonality are included. A number of conclusions can be drawn from these findings and the presented case. First, both monoclonal and biclonal lymphomas can present in the clinical form of a RS; among published cases, biclonal cases are predominant. Second, expression of a common immunoglobulin isotype in CLL and LCL cells is not sufficient to conclude monoclonality in RS. Only four out of 11 cases which could be shown to be biclonal by analysis of gene rearrangement presented with different immunoglobulin isotypes in CLL and LCL cells. Third, except for one report of a loss of IgD expression in the LCL (Bertoli *et al.*, 1987), a *de novo* expression or loss of differentiation antigens has not been described in monoclonal cases of RS. The fact that only discrepancies concerning CD5 expression (four cases) and CD21 expression (one case) were reported in biclonal RS tumours is probably due to the quite limited number of differentiation antigens studied in the published cases.

Althouth CLL and CBp cells in patient HK both originated from one B cell clone having undergone immuno-

**Table I** Immunophenotypic marker studies on HK tumour samples

| *Antigen* | *Clone* | *Reactivity in* CLL | CBp |
|---|---|---|---|
| CD2 | OKT11 | − | − |
| CD3 | Leu4 | − | − |
| CD4 | OKT4 | − | − |
| CD5 | OKT1 | + | − |
| CD8 | OKT8 | − | − |
| CD38 | OKT10 | − | + |
| CD10 | J5 | − | − |
| CD19 | HD37[a] | + | − |
| CD20 | 1F5 | + | + |
| CD21 | B2 | − | − |
| CD22 | HD39[a] | + | − |
| CD23 | HD50[a] | − | + |
| CD24 | VIB-E3 | + | − |
| CD37 | HD28[a] | + | + |
| CD40 | G28-5 | + | + |
| CD53 | HD77[a] | + | + |
| HLA-A,B,C | W6/32 | + | + |
| HLA-DR | ISCR3 | + | + |
| HLA-DP | B7/21 | + | + |
| HLA-DQ | Tü22 | − | + |
| Inv. Chain | VIC-Y1 | + | + |
| CD11c | LeuM5 | − | − |
| CD13 | My7 | − | − |
| CD14 | BEAR2 | − | − |
| CD15 | LeuM1 | − | − |
| CD30 | Ki-1 | − | − |
| | FMC7 | − | − |
| Vimentin | V9 | + | − |
| gp80 | G28-8 | + | + |
| HK-Idiotype | AHK154.3[a] | + | + |
| HK-Idiotype | AHK120.8[a] | + | + |
| Lambda | 1-155-2 | − | − |
| kappa | NHV361[a] | + | + |
| IgM | NLH205[a] | + | + |
| IgG | 8a4 | − | − |

[a]Antibodies produced in our laboratory. MAbs to differentiation antigens are described in 'Leucocyte Typing IV' (Knapp *et al.*, 1989).

**Figure 2** Immunostained serial sections of bone marrow removed at autopsy (× 90). **a**, CD24 (VIB-E3) selectivity reacts with small lymphoid cells corresponding to the B-CLL subpopulation (scale bar = 50 μm); **b**, CD23 (HD50) selectively stains the centroblastic subset of the neoplastic population; **c**, CD37 (HD28) is expressed on both subsets of tumour cells; **d**, likewise, anti-idiotypic monoclonal antibody AHK154 reacts with both B-CLL and CBp cells; **e**, HLA-DR antigens are expressed on small and large tumour cells as determined by MAb ISCR3; **f**, In contrast, HLA-DQ antigen expression is restricted to the centroblastic subset, as shown by reactivity with MAb Tü22.

globulin gene heavy chain rearrangement, they displayed a distinct pattern of differentiation antigen expression. The phenotype of CLL cells with expression of pan-B markers CD19 and CD20, B-restricted markers CD22 and CD24 and expression of CD5 is in accordance with the data obtained in larger studies (Schuurman *et al.*, 1987). CBp cells displayed an immunophenotype (vimentin$^-$, CD21$^-$, CD30$^-$, CD38$^+$) which is highly suggestive of a follicular centre cell stage of maturation. Thus, morphological as well as immunophenotypic criteria indicate a transition from chronic lymphatic leukaemia to a polymorphic centroblastic lymphoma.

The transition from CLL to CBp may have been caused by mutational events not involving the immunoglobulin heavy chain variable region. Translocations juxtaposing the immunoglobulin light chain or heavy chain genes to a number of oncogenes are commonly found in certain types of B cell lymphomas. Such mutations were shown to lead to a deregulation of the oncogenes and may, in part, be detectable as rearrangements of the translocated gene sequences. Translocations joining the bcl-2 oncogene to immunoglobulin genes are found in 85 per cent of lymphomas with follicular centre cell type and, in very rare cases, in CLL (Adachi *et al.*, 1989) and are thought to give rise mainly to indolent, slowly progressive lymphomas with nodular architecture (Yunis *et al.*, 1987). Translocations involving the c-*myc* oncogene together with immunoglobulin genes are found in the highly malignant Burkitt lymphomas and are associated with a high rate of cell proliferation (Taub *et al.*, 1982). In addition,

**Figure 3** Ig heavy chain gene rearrangement in HK tumour tissue. 10 μg DNA, digested with BamHI and HindIII, was hybridised with a 5.5 kb genomic fragment from the human immunoglobulin heavy chain joining region. Filled arrows indicate rearranged bands, the open arrow marks the germline band. DNA samples were prepared from A: HK spleen, B: HT 29 (human colon carcinoma line), C: HK lymph node 1, D: buffy coat from a healthy volunteer, E: HK lymph node 2, F: HK-PBL from 1985, G: Jurkat T lymphoma line, H: HK bone marrow.

follicular lymphomas with a bcl-2 translocation may convert into highly malignant lymphomas upon further translocational events involving the c-*myc* gene (De Jong, 1988). Therefore, we asked whether a translocation of c-*myc* into an immunoglobulin gene locus might have caused transformation of CLL cells to a highly malignant lymphoma with a high rate of proliferation. Owing to the lack of viable tumour cells from the clinical stage of RS, a cytogenetic analysis could not be done. Therefore, genomic DNA from CLL and

**Table II** Clonality in published cases of Richter's syndrome

| *Authors* | *Cases* | *Ig Isotype in CLL/LCL* | *Ig gene re-arrangement* | *Phenotypic differences (losses in LCL)* |
|---|---|---|---|---|
| Splinter | 1 | μλ/μκ | N.T. | N.T. |
| Delsol | 1 | μκ/μλ | N.T. | N.T. |
| Chan | 4[a] | 1 identical<br>3 divergent | N.T. | 3/4: CD5 |
| Michiels | 1 | μκ/μκ | monoclonal | No |
| Bertoli | 1 | μλ/μλ | monoclonal | IgD |
| Ostrowski | 1 | μλ/– | biclonal | No |
| McDonnell | 1 | μκ/μλ | biclonal | CD21 |
| Van Dongen | 1 | μλ/μκ | biclonal | CD5 |
| Sklar | 3[a] | 2 identical<br>1 divergent | 3 biclonal | N.T. |
| Siegelman | 7[a] | 6 identical<br>1 divergent | 5 biclonal<br>2 monoclonal | N.T. |

[a]Including B cell lymphomas with two histological types without involvement of CLL.

CBp tumour cells was analysed for a possible rearrangement of the c-*myc* gene which might indicate a translocation. Using a 300 bp PstI-fragment from the second exon of the c-*myc* gene, identical patterns of restriction enzyme fragments were obtained when DNA from both histological subsets of HK lymphoma and DNA from control normal lymphocytes was digested with PstI, SstI, SmaI and XhoI enzymes (data not shown). Thus no evidence for a translocation involving c-*myc* or other genetic events resulting in a clonal evolution towards high grade malignancy can be provided in the case presented.

Support was provided by the Tumorzentum Heidelberg Mannheim.

### References

ADACHI, M., COSSMAN, J., LONGO, D., CROCE, C.M. & TSUJIMOTO, Y. (1989). Variant translocation of the bcl-2 gene to the immunoglobulin lambda light chain in chronic lymphocytic leukemia. *Proc. Natl Acad. Sci. USA*, **86**, 2771.

ARNOLD, A., COSSMAN, J., BAKSHI, A., JAFFE, E.S., WALDMANN, T.S. & KORSMEYER, S.J. (1983). Immunoglobulin-gene rearrangements as unique clonal markers in human lymphoid neoplasms. *N. Engl. J. Med.*, **26**, 1594.

BERTOLI, L.F., KUBAGAWA, H., BORZILLO, G.V. & 5 others (1987). Analysis with anti-idiotype antibody of a patient with CLL and large cell lymphoma. *Blood*, **70**, 45.

CHAN, W.C. & DEKMEZIAN, R. (1986). Phenotypic changes in large cell transformation of small cell lymphoid malignancies. *Cancer*, **57**, 1971.

DE JONG, D., VOETDIJK, B.M.H., BEVERSTOCK, G.C., VAN OMMEN, G.J.B., WILLEMZE, R. & KLUIN, P.M. (1988). Activation of the c-myc oncogene in a precursor B cell blast crisis of follicular lymphoma, presenting as composite lymphoma. *N. Engl. J. Med.*, **318**, 1373.

DELSOL, G., LAURENT, G., KUHLEIN, E., FAMILIADES, J., RIGAL, F. & PRIS, J. (1981). Richter's syndrome. Evidence for the clonal origin of the two proliferations. *Am. J. Clin. Pathol.*, **76**, 308.

KNAPP, W., DÖRKEN, B., GILKS, W.R. & 4 others (eds) (1989). *Leucocyte typing IV*. Oxford; Oxford University Press.

LENNERT, K., MOLIN, N., STEIN, H., KAISERLING, E. & MÜLLER-HERMELINK, H.K. (1978). Malignant lymphomas other than Hodgkin's disease. Springer-Verlag: Berlin.

MAYUMI, M., JUBAGAWA, H., OMUA, G.A., GATHINGS, W.E., KEARNEY, J.F. & COOPER, M.D. (1982). Studies on the clonal origin of human B cell leukemia using monoclonal anti-idiotype antibodies. *J. Immunol.*, **129**, 904.

MCDONNELL, J.M., BESCHORNER, W.E., STAAL, S.P., SPIVAK, J.L. & MANN, R.B. (1986). Richter's syndrome with two different B cell clones. *Cancer*, **58**, 2031.

MICHIELS, J.J., VAN DONGEN, J.J.M., HAGEMEIJER, A. & 6 others (1989). Richter's syndrome with identical immunoglobulin gene rearrangements in the chronic lymphocytic leukemia and the supervening Non-Hodgkin's lymphoma. *Leukemia*, **3**, 819.

MÖLLER, P., MATTHAEI-MAURER, D.U., HOFMANN, W.J., DÖRKEN, B. & MOLDENHAUER, G. (1989). Immunophenotypic similarities of mediastinal clear-cell lymphoma and sinusoidal (monocytoid) B cells. *Int. J. Cancer*, **43**, 10.

MOLDENHAUER, G., DÖRKEN, B., PEZZUTTTO, A., SCHWARTZ, R., KNOPST, J. & HÄMMERLING, G.J. (1983). Monitoring of a human B cell lymphoma with monoclonal anti-idiotype antibodies. In: Peeters, H. (ed.), *Protides of the biological fluids*. Pergamon Press: Oxford. **32**, 867.

OSTROWSKI, M., MINDEN, M., WANG, C. & BAILEY, D. (1989). Immunophenotypic and gene probe analysis of a case of Richter's syndrome. *Am. J. Clin. Pathol.*, **91**, 215.

SCHUURMAN, H.J., VAN BAARLEN, J., HUPPES, W., LAM, B.W., VERDONCK, L.F. & VAN UNNIK, J.A.M. (1987). Immunophenotyping of Non-Hodgkin's lymphoma: lack of correlation between immunophenotype and cell morphology. *Am. J. Pathol.*, **129**, 140.

SIEGELMAN, M.H., CLEARY, M.L., WARNKE, R. & SKLAR, J. (1985). Frequent biclonality and Ig gene alterations among B cell lymphomas that show multiple histologic forms. *J. Exp. Med.*, **161**, 850.

SKLAR, J., CLEARY, M.L., THIELEMANNS, K., GRALOW, J., WARNKE, R. & LEVY, R. (1984). Biclonal B cell lymphoma. *N. Engl. J. Med.*, **311**, 20.

SPLINTER, T.A.W., BOM-VAN NOORLOOS, A. & HEERDE, P. (1978). CLL and diffuse histiocytic lymphoma in one patient: clonal proliferation of two different B cells. *Scand. J. Haematol.*, **20**, 29.

TAUB, R., KIRSCH, I., MORTON, C. & 5 others (1982). Translocation of the c-myc gene into the immunoglobulin heavy chain locus in human Burkitt lymphoma and murine plasmocytoma cells. *Proc. Natl Acad. Sci. USA*, **79**, 7837.

TRUMP, D.L., MANN, R.B., PHELPS, R., ROBERTS, H. & LOCKARD CONLEY, C. (1980). Richter's syndrome: diffuse histiocytic lymphoma in patients with CLL: a report of five cases and review of the literature. *Am. J. Med.*, **68**, 539.

VAN DONGEN, J.J.M., HOOIJKAAS, H., MICHIELS, J.J. & 6 others (1984). Richter's syndrome with different immunoglobulin light chains and different heavy chain rearrangements. *Blood*, **64**, 571.

YUNIS, J.Y., FRIZZERA, G., OKEN, M.M., MCKENNA, J., THEOLOGIDES, A. & ARNESEN, M. (1987). Multiple recurrent genomic defects in follicular lymphoma: a possible model for cancer. *N. Engl. J. Med.*, **316**, 79.